# *Azolla pinnata* mitigates pendimethalin induced immunotoxicity, oxidative stress and histopathological changes in *Oreochromis niloticus*

**DOI:** 10.1038/s41598-025-96757-2

**Published:** 2025-05-09

**Authors:** Nagwa I. S. Abu-Zahra, Mofeed Gouda, Mohamed M. Elseify, Mona E. Abass, Mohammed S. El-Gohary, Eman T. El-sokary

**Affiliations:** 1 Fish Diseases Unit, Kafrelsheikh Provincial Lab, Animal Health Research Institute (AHRI), Agriculture Research Center (ARC), Giza, Egypt; 2 Pathology Unit, Kafrelsheikh Provincial Lab, Animal Health Research Institute (AHRI), Agriculture Research Center (ARC), Giza, Egypt; 3 Immunology Unit, Kafrelsheikh Provincial Lab, Animal Health Research Institute (AHRI), Agriculture Research Center (ARC), Giza, Egypt; 4 Biochemistry, Nutritional Deficiency Diseases and Toxicology Unit, Kafrelsheikh Provincial Lab, Animal Health Research Institute, Agricultural Research Center (ARC), Giza, Egypt

**Keywords:** *Azolla pinnata*, Histopathology, *Oreochromis niloticus*, Pendimethalin, RBC morphology, Biochemistry, Immunology, Microbiology, Physiology, Environmental sciences

## Abstract

Aquatic animals face multiple threats, including pesticides, heavy metals, and other environmental pollutants, risking their health and survival. Limited bioremediation studies have been conducted on the detrimental impacts of herbicides on fish. This study focused on the impact of the herbicide pendimethalin (PD) on *Oreochromis niloticus* and assessed the protective role of *Azolla pinnata* (AZ), an aquatic fern known for its phytoremediation and antioxidant properties. *O. niloticus* (*n* = 270, 34.17 ± 2.41 g) were divided into six groups in triplicate: the control (CTR), AZ-supplemented (125 g/kg diet), PD-exposed (0.5 and 1 mg PD/L), and PD-exposed with AZ supplementation (0.5 and 1 mg PD/L + AZ) groups for 28 days. PD exposure caused substantial reductions in growth performance and hematological indices (hemoglobin concentration (Hb) and red blood cell (RBC) count), with significant increases in white blood cell (WBC) count. Oxidative damage from PD exposure was evidenced by decreased superoxide dismutase (SOD) activity and acetylcholinesterase (AChE) levels, along with increased malondialdehyde (MDA) levels in hepatic and gill tissues. PD-exposed fish also presented reduced phagocytic activity (PA) and index (PI), along with decreased lysozyme activity and resistance to *Pseudomonas aeruginosa* infection. Additionally, hepatic and renal damage markers (AST, ALT, ALP, urea, and creatinine) and stress indicators (cortisol and glucose) were notably elevated. Severe tissue and cellular damage further highlight PD-induced damage. AZ supplementation had a protective effect, almost restoring normal growth performance, hematological parameters, and antioxidant defenses. AZ improved SOD and AChE activity and reduced MDA levels, mitigating oxidative damage. AZ also improved immune responses, restoring PA, PI, and lysozyme activity and bacterial resistance. Furthermore, AZ alleviated hepatic and renal damage, normalized stress markers, and mitigated tissue and morphological abnormalities, preserving tissue integrity. This study underscores the potential of dietary AZ supplementation (12.5%) as a growth promoter, antioxidant, and immunostimulant in aquaculture, effectively enhancing resistance to environmental toxicants and bacterial infections.

## Introduction

Aquatic organisms are exposed to various environmental hazards, such as heavy metals, trace elements, pesticides, and microbial contaminants, which can significantly affect their health and survival^1^. Among these, herbicides are widely used in agriculture to control weeds, but their improper application can lead to contamination of aquatic ecosystems, affecting nontarget organisms, particularly fish. In Egypt, pendimethalin (PD) is a commonly used preemergent herbicide for managing broadleaf weeds and grasses in various crops^2^. Owing to its extensive application, PD can be detected in the environment, particularly in agricultural soils and water bodies^2^. PD is a systemic herbicide that contaminates aquatic systems and soil through drainage and leakage, persisting in the environment because of its bioaccumulative nature^3^. It undergoes degradation through photodecomposition, volatilization, and biodegradation, but its stability is influenced by environmental factors, making it a potential ecological risk, particularly for aquatic organisms^4^.

Fish must engage in various defense mechanisms to combat the damaging effects of PD, which in turn alter their metabolism and impact their immunity, reproduction, growth, and survival^5^. PD can also cause oxidative stress in fish, damaging vital cellular components, such as lipids, proteins, and DNA^3,6^. The no observed effect concentration (NOEC) of PD is 43 µg/L for fish and 11 µg/L for algae^7^. Given the established toxicity of PD in animal models, investigating its toxic impacts on fish is crucial to better understand its specific effects on this group of nontarget organisms. Fish are integral to aquatic ecosystems, and their health directly influences the overall health of these environments. Understanding the toxicological impacts of PD on fish will help inform strategies for mitigating the adverse effects of herbicide contamination in aquatic ecosystems, ultimately contributing to the protection and preservation of aquatic biodiversity.

The use of plant bioactive compounds has been extensively studied in aquatic animals for their ability to enhance liver and intestinal health, mitigate toxic hazards, and improve antioxidant and immune functions. These natural compounds show significant potential in reducing the harmful effects of environmental pollutants, xenobiotics, and other toxicants, thereby supporting overall physiological stability^8–10^. Nutritional interventions with plant bioactive compounds have also been shown to improve fish flesh quality, immune response, and antioxidant capacity, highlighting their role in sustainable aquaculture^10,11^. Studies further demonstrate the effectiveness of medicinal herbs in protecting fish from pollution, aiding detoxification, and promoting overall health and growth^6,12,13^. Additionally, plant bioactive compounds may reduce dependency on synthetic drugs, which can negatively impact both fish and their ecosystems. However, their specific application in enhancing resistance to PD toxicity in fish remains limited.

*Azolla pinnata* (AZ) has been efficiently employed as a feed additive for tilapia culture^14^. AZ may be a useful feed additive in the future that will help farmers achieve sustainability and is both ecologically and economically practical^14^. Additionally, AZ is a feed component that does not compete with human food sources and has low land requirements, high productivity, low manufacturing costs, and abundant water bodies^15^. Because of its superior purification effects from direct contact with contaminated water, higher pollutant uptake capacity, rapid growth and greater biomass production, AZ is more suitable for wastewater treatment^16^. Furthermore, they substantially contribute to the structural and functional characteristics of aquatic environments by controlling the nutrient cycle, oxygen balance, and heavy metal accumulation and modifying water quality^17^.

Bioactive compounds in AZ species, including tannins, polyphenols, flavonoids, and alkaloids, exhibit significant antimicrobial properties by disrupting microbial metabolism and interacting with cell walls and membranes^18,19^. AZ-derived polysaccharides, such as cellulose and hemicellulose, are recognized for their antioxidant, immunomodulatory, and detoxification effects^20,21^. These polysaccharides enhance enzymatic antioxidant defenses by increasing superoxide dismutase (SOD) and glutathione (GSH) activities while reducing malondialdehyde (MDA) levels, protecting aquatic organisms from oxidative damage^20^. Additionally, they regulate key cellular pathways, such as nuclear factor-kappa B (NF-kB), contributing to anti-inflammatory and hepatoprotective effects^10,21^. These properties make AZ a promising candidate for reducing toxic hazards, such as PD contamination, while supporting fish health and resilience.

One of the most commonly used freshwater fish in toxicological research is *O. niloticus* because of its rapid growth, ease of adaptability to commercial feeds, and tolerance to injuries and diseases^22^. Several markers provide insights into fish health under herbicide and environmental stress. Growth performance and hematological indices serve as key indicators of overall health, with deviations potentially reflecting physiological stress, metabolic dysfunctions, or immune suppression^23^. Immune parameters, including phagocytic activity, lysozyme activity, and resistance to pathogens, are used to evaluate immune competence^11^. Evaluating these parameters offers a comprehensive understanding of fish health and the effectiveness of dietary interventions.

This study aims to address this knowledge gap by evaluating the toxicological impact of PD on fish, with a focus on oxidative stress, metabolic disturbances, and adaptive defense responses. By assessing long-term exposure across different concentrations, our findings contribute to a better understanding of PD-induced toxicity and provide critical data for environmental risk assessment and aquatic health management. While studies on the use of AZ for bioremediation are limited, our study sought to explore its effectiveness in mitigating the detrimental effects of PD on fish. We hypothesized that AZ could serve as a natural, cost-effective bioremediation agent, reducing the toxic impacts of PD on aquatic organisms. We anticipated that PD exposure would lead to oxidative stress and impair antioxidant defense mechanisms. The novel expectation was that dietary AZ supplementation would increase the antioxidant capacity and reduce oxidative stress in fish. Given the immunomodulatory properties of AZ, we hypothesized that it could alleviate PD-induced immunotoxic effects, restore hematological indices and histological changes, and improve immune responses, thereby increasing the resistance of fish to *P. aeruginosa* infection. Another novel aspect of our study was the assessment of the dose-dependent protective effects of AZ. We aimed to determine whether different PD concentrations (0.5 and 1 mg/L) could be effectively countered by consistent dietary supplementation with AZ (125 g/kg diet), highlighting the practical implications for aquaculture practices.

## Materials and methods

### Chemicals

This study used a commercial PD formulation from Stomp^®^ Extra 45.5% CS, BASF PLC, Egypt (455 g/L PD). Based on previously reported 96 h LC_50_ values for *O. niloticus* (5.15 mg/L^12^ and 4.92 mg/L^24^), two sublethal concentrations, 0.5 mg/L (1/10 LC_50_) and 1 mg/L (1/5 LC_50_), were selected.

To prepare the test solutions, the required amount of PD was first dissolved in distilled water in a volumetric jar to create a stock solution. No vehicle was used in this process to ensure the purity of the test compound. The stock solution was then diluted with dechlorinated aquarium water to achieve the desired concentrations of 0.5 mg/L and 1 mg/L PD. The dilution process involved adding a calculated volume of the stock solution to a known volume of tank water, ensuring accurate concentration levels. The water in the aquaria was continuously aerated to maintain homogeneity. Throughout the experiment, the concentrations were periodically verified via a spectrophotometer to ensure consistency. The chemical ratio in the tank water was controlled by maintaining these concentrations throughout the study. Specifically, the ratio of PD in the tank water was either 0.5 mg/L or 1 mg/L, depending on the test conditions. The following formula was used for dilution:

$$\:\text{C}1\text{}\text{V}1\text{}=\text{C}2\text{}\text{V}2$$

where C1 is the concentration of the stock solution, V1 is the volume of stock solution needed, C2 is the desired concentration in the tank, and V2 is the total volume of water in the tank.

The nominal concentrations were set by using the aforementioned dilution formula to achieve the desired levels of PD in the tank water, ensuring that the concentrations were accurate and consistent throughout the experiment.

### Feed preparation

The results of the proximate analysis of the fish diets are presented in Table 1. A commercially available fish diet was used. The AZ was acquired from a private farm in Kafrelsheikh Province, Egypt, sun-dried and used in the fish diet as a powder. Nutrient analysis of AZ (Table 1) was performed via a standard method^25^. Two diets were prepared: a control (CTR diet) and an AZ diet. The AZ diet was made by adding AZ to the CTR diet in proportions of 12.5% for the treatments (AZ, 0.5 PD + AZ, and 1 PD + AZ), as shown in Table 1. The pellet feed was ground, and then 125 g of AZ/kg diet was thoroughly mixed with the diet for ½ an hour to create a dough. Prior to being formed into pellets with a diameter of 2 mm, every dough was run through a grinder. The feedstuffs were oven dried for 24 h at 50 °C before being stored at − 2 °C. The CTR diet was made in the same manner and devoid of any additives.

**Table 1:** Proximate analysis of the experimental diets and nutrient composition of AZ (in % dry matter).

Chemical composition of CTR diet			Nutrient composition of AZ	
Nutrient %	CTR diet	12.5% AZ diet	Nutrient %	AZ
Dry matter	91.73	93.25	Dry matter	100
Moisture	8.27	6.75	Moisture	0.00
Crude protein	27	27.76	Crude protein	34.33
Crude fat	5	4.82	Crude fat	3.22
Ash	5.8	6.88	Ash	14.64
Crude fiber	4.2	5.08	Cellulose	17.96
Nitrogen free extract (NFE)	50	49.52	Hemicellulose	11.93
Gross energy (GE) MJ	17.6	17.33	Lignin	25.69
			ADF	43.65
			NDF	55.58

The CTR diet was purchased from Aller-Aqua Company, Egypt.

The ingredients of the CTR diet listed on the producer label are corn gluten, DDGS, fish meal, fish oil, maize, monocalcium phosphate, rice bran, salt, soya oil, soya, sunflower meal, vitamins and minerals, wheat middling, and wheat products.

*ADF* acid detergent fiber, *NDF* neutral detergent fiber.

### Culture facilities and experimental setup

*O. niloticus* (*n* = 270, 34.17 ± 2.41 g, approximately 5–6 months old) were acquired from a local farm in Kafrelsheikh Province, Egypt. During a 14-day adaptation period with a 12 h photoperiod, no clinical signs were detected in the fish. The aquaria (40 × 40 × 50 cm) were filled with dechlorinated tap water from storage tanks. The aquaria were continuously aerated, and the debris along with two-thirds of the aquarium water was siphoned daily. The fish were distributed haphazardly into 6 groups (Table 2) in three replicates (*n* = 45/group, 15/replicate) and fed 2 times/day at 09:00 and 14:00. The 1st group was the CTR group. The 2nd group was fed a diet containing AZ (12.5%). The 3rd and 4th groups were exposed to 0.5 and 1 mg PD/L, respectively. The 5th and 6th groups were exposed to 0.5 and 1 mg PD/L, respectively, and each group was fed a diet containing AZ (12.5%) for 28 days. Throughout the experiment, a semistatic system was maintained, where clean water with the same concentrations of PD was added daily to substitute for the water in the aquaria. This approach ensured that the water quality was consistently maintained. Every day, mortality, feed intake, and external signs were recorded.

During the feeding trial and experimental toxicity test, the fish were fed at a rate of 3% of their live body weight. Each feeding session was followed by a visual evaluation of the feed intake. Fish were weighed every 2 weeks to calculate weight gain differences, and feed intake was estimated by the biomass in each aquarium. The following equation was used to estimate the survival rate (SR%):

$$\:\text{S}\text{R}\text{\%}=\frac{\:\:\text{F}\text{i}\text{n}\text{a}\text{l}\:\text{n}\text{u}\text{m}\text{b}\text{e}\text{r}\:\text{o}\text{f}\:\text{f}\text{i}\text{s}\text{h}\:}{\:\:\text{I}\text{n}\text{t}\text{i}\text{a}\text{l}\:\text{n}\text{u}\text{m}\text{b}\text{e}\text{r}\text{o}\text{f}\:\text{f}\text{i}\text{s}\text{h}\:\:}\:\times\:\:100$$

**Table 2:** Experimental design of the fish groups.

Fish group	No. fish	Type of treatment	Treatment code	Tested concentration of PD	AZ
1	45	Control	CTR	×	×
2	45	Fed on AZ	AZ	×	125 g/kg feed (12.5%)
3	45	Exposed to 0.5 mg/L PD	0.5 PD	0.5 mg/L	×
4	45	Exposed to 1 mg/L PD	1 PD	1 mg/L	×
5	45	Exposed to 0.5 mg/L PD and fed on AZ	0.5 PD + AZ	0.5 mg/L	125 g/kg feed (12.5%)
6	45	Exposed to 1 mg/L PD and fed on AZ	1 PD + AZ	1 mg/L	125 g/kg feed (12.5%)

### Water quality parameters

The quality parameters of the water were regularly checked via a water analysis device. During the trial, the pH, dissolved oxygen content, and temperature were maintained at 7.2 ± 0.2, 7.3 ± 0.2 mg/L, and 26 °C ± 1 °C, respectively. Once a week, the alkalinity was checked by titration with H2SO4 until the pH reached 4.5^26^. The values of nitrite (NO2-N) and ammonia (NH3-N) were determined via a water analysis photometer (MultiDirectLovibond).

### Growth performance

At the end of the trial (28 days), the fish were weighed to obtain the final body weight (g) and other growth indices according to the following equations^27^:

$$\:\text{W}\text{G}\:\left(\text{g}\right)=\text{W}1-\text{W}0$$

$$\:\text{G}\:\text{\%}=\frac{\text{W}\text{G}}{\text{W}0}\times\:100\:$$

$$\:\text{F}\text{C}\text{R}\:=\frac{\text{T}\text{F}\text{I},\:\text{g}}{\text{W}\text{G},\:\text{g}}$$

$$\:\text{S}\text{G}\text{R}=\frac{\text{l}\text{n}\:\text{W}1\:\left(\text{g}\right)-\text{l}\text{n}\text{W}0\:\left(\text{g}\right)}{\text{T}\:}\times\:100$$

$$\:\text{P}\text{E}\text{R}\:=\frac{\text{W}\text{G}\:,\text{g}}{\:\text{P}\text{I}\:,\text{g}}\:\:$$

W0: initial weight (g), W1: final weight (g), WG: weight gain (g), G%: gain%, FCR: feed conversion ratio, SGR: specific growth rate, TFI: total feed intake (g), T: period in days, PER: protein efficiency ratio, PI: protein intake (g).

### Sampling

Following the 28-day experimental trial, fish were subjected to a 24-hour starvation period prior to sampling. Euthanasia was performed by submerging the fish in an anesthetic solution containing 40 mg/L clove oil^28^, ensuring minimal distress. Once the fish were fully anesthetized and showed no opercular movement, they were sampled. This method ensured humane handling during the subsequent sampling process. Blood samples were collected from the caudal vessels and divided into two sets. The first set, intended for hematological parameter analysis (*n* = 9/treatment), was collected in EDTA tubes and stored at 4 °C for same-day analysis. The second set, designated for serum analysis (*n* = 9/treatment), was collected without anticoagulants. After centrifugation at 3000 rpm for 5 min, the serum was transferred to Eppendorf tubes and stored at − 20 °C for analysis within two weeks. After euthanasia, tissue samples (liver, gills, and middle intestinal portions, *n* = 9/treatment) were carefully dissected. For histological investigations, the tissues were preserved in 10% formalin. Additional tissue samples (liver and gills, *n* = 9/treatment) were stored at − 20 °C for antioxidant activity assays.

### Hematological indices and RBC abnormality evaluations

The RBC count (×10^6^/mm^2^) and WBC count (×10^3^/mm^2^) were estimated via a Neubaur hemocytometer with modifications. Hb (g/dL) and PCV (%) were assessed via the cyanmethemoglobin and microhematocrit methods, respectively. The formulas mentioned by Briggs and Bain^29^ were used to determine blood indices.

$$\:\text{M}\text{C}\text{V}\left(\text{f}\text{L}\right)=\frac{\text{P}\text{C}\text{V}}{\text{R}\text{B}\text{C}\text{s}}\times\:10$$

$$\:\text{M}\text{C}\text{H}\:\left(\text{p}\text{g}\right)=\frac{\left(\text{H}\text{b},\:\text{g}/\text{d}\text{L}\right)}{\text{R}\text{B}\text{C}\text{s}}\times\:10$$

$$\:\text{M}\text{C}\text{H}\text{C}\:(\text{g}/\text{d}\text{L})=\frac{\left(\text{H}\text{b},\:\text{g}/\text{d}\text{L}\right)}{\text{P}\text{C}\text{V}}\times\:100$$

Differential leukocyte counts (x10^2^/mm^2^) were evaluated by making thin blood films on sterile slides that were stained with a modified Wright’s stain after being allowed to dry. RBC abnormalities were evaluated following the method described by Hussain et al.^30^ with slight amendments. Using a Leica DM 500 phase-contrast microscope connected to an ICC50W camera, slides were chosen, coded, and evaluated for morphological changes on the basis of the staining quality.

### Preparation of bacteria for phagocytosis

*Escherichia coli* strain B (Sigma‒Aldrich, product number EC11303) at a concentration of 10^6^ CFU/mL was centrifuged at 8000 rpm for 30 min and rinsed twice with physiological saline. After being concentrated, the bacterial culture was resuspended in saline. Then, in sterile 12 × 75-mm culture tubes, 1/2 mL of fresh EDTA blood and 1/4 mL of *E. coli* suspension (7.1 × 10^6^ CFU/mL) were thoroughly mixed for the phagocytic assay. The tubes were shaken every 10 min and kept at 28 °C for 30 min before being centrifuged at 1000 rpm for 5 min. The supernatant was discarded, and the upper layer of the precipitate was utilized to make blood smears and stained with Giemsa. Leukocyte engulfment percentages were calculated on the basis of the total number of leukocytes in the smear obtained from the phagocytic assay.

$$\:\text{P}\text{A}\:\text{\%}\:\left(\text{P}\text{h}\text{a}\text{g}\text{o}\text{c}\text{y}\text{t}\text{i}\text{c}\:\text{a}\text{c}\text{t}\text{i}\text{v}\text{i}\text{t}\text{y}\right)=\text{\%}\:\:\text{p}\text{h}\text{a}\text{g}\text{o}\text{c}\text{y}\text{t}\text{i}\text{c}\:\text{c}\text{e}\text{l}\text{l}\text{s}\:\text{c}\text{o}\text{n}\text{t}\text{a}\text{i}\text{n}\text{i}\text{n}\text{g}\:\text{b}\text{a}\text{c}\text{t}\text{e}\text{r}\text{i}\text{a}\:$$

$$\:\text{P}\text{I}\:\text{N}\text{o}\:\left(\text{P}\text{h}\text{a}\text{g}\text{o}\text{c}\text{y}\text{t}\text{i}\text{c}\:\text{i}\text{n}\text{d}\text{e}\text{x}\right)=\frac{\text{T}\text{o}\text{t}\text{a}\text{l}\:\text{n}\text{u}\text{m}\text{b}\text{e}\text{r}\:\text{o}\text{f}\:\text{p}\text{h}\text{a}\text{g}\text{o}\text{c}\text{y}\text{t}\text{i}\text{z}\text{e}\text{d}\:\text{b}\text{a}\text{c}\text{t}\text{e}\text{r}\text{i}\text{a}\text{l}\:\text{c}\text{e}\text{l}\text{l}\text{s}}{\text{P}\text{h}\text{a}\text{g}\text{o}\text{c}\text{y}\text{t}\text{i}\text{c}\:\text{c}\text{e}\text{l}\text{l}\:\text{c}\text{o}\text{u}\text{n}\text{t}}$$

### Lysozyme activity

In accordance with Abo-Al-Ela et al.^31^, a 1% agarose gel plate was prepared by dissolving agarose in an appropriate buffer solution and allowing it to solidify in a petri dish. A total of 500 mg/L *Micrococcus lysodeikticus* was suspended in the buffer solution. The prepared *M. lysodeikticus* suspension was spread evenly over the surface of the agarose gel plate. Wells were created in the agarose gel via a sterile cork borer, and 50 µL of each serum sample was added to each well. The agarose gel plate was incubated at 37 °C for 18–24 h to allow the lysozyme to diffuse and lyse the bacterial cells. The width of the clear lysis zones around each well was measured via a Vernier caliper. The following formula was used to calculate the activity via logarithmic regression:

$$\:\text{Y}=\text{A}+\text{B}\text{log}\text{x}$$

where Y is the width of the lysis zone, X is the activity of lysozyme, and A and B are constants determined by logarithmic regression.

### Biochemical parameters

Albumin and total serum protein were measured in accordance with the methods of Zheng et al.^32^. The difference between the serum albumin (ALB) concentration and the total protein concentration was used to compute the globulin concentration. The activities of AST, ALP, ALT, urea, creatinine, and glucose were assessed calorimetrically^33^ via a spectrophotometer. According to the parameters evaluated, the absorbency of the sample under examination was measured at a suitable wavelength between 340 and 546 nm. Serum AChE and cortisol levels were estimated following the manufacturer’s instructions.

Briefly, 50 µL of serum was added to each well of a microtiter plate containing 50 µL of biotin-conjugated AChE, which was subsequently incubated for 60 min at 37 °C. When the precoated antibody specific for AChE was used, a competitive inhibition process was initiated between the AChE of the samples and biotin-conjugated AChE. The amount of antibody bound by biotin-conjugated AChE decreases with increasing AChE levels in samples. Following washing, the wells were filled with avidin-conjugated horseradish peroxidase (HRP). When the substrate mixture was added, the color changed in contrast to the sample’s AChE content. The color intensity was measured after the color change stopped.

To estimate cortisol, 50 µL of serum and 50 µL of a cortisol-specific antibody were added to the microtiter plate wells, and the plates were then incubated for 40 min at 37 °C. A goat anti-rabbit antibody conjugated with HRP (100 µL) was added. Serum cortisol and precoated cortisol initiate competitive inhibition reactions. When a substrate solution is applied to the wells, the color changes in direct proportion to the sample’s cortisol content. The color intensity was measured after the color change stopped.

Serum electrolytes (Na^+^, K^+^, and Cl^-^) were estimated via an ST-200 Plus Electrolyte Analyzer (SENSA CORE, India). Information on the kits and reagents used to measure the serum biochemical parameters is provided in Table 3.

**Table 3:** Details regarding the kits used to estimate the serum biochemical parameters.

Parameters	Kits and reagents used	CAT no.
ALT	Roche Diagnostic, Indianapolis, IN, USA	05850797188
AST	Roche Diagnostic, Indianapolis, IN, USA	05850819188
ALP	Roche Diagnostic, Indianapolis, IN, USA	03333701190
Glucose	Biomed, Egypt	GLU-109,240
Creatinine	Abcam, UK	ab65340
Urea	Spectrum, Egypt	318 001
Total protein	Spinreact, Spain	MD1001291
Albumen (ALB)	Spinreact, Spain	MD1001020
Cortisol	Eliza kits from Cusabio Technology LLC, USA (sensitivity: 1.56 ng/mL)	CSB-E08487f
ACHE	Eliza kits from Cusabio Technology LLC, USA (sensitivity: 2.5 ng/mL)	CSB-E17001Fh

### Tissue extraction and antioxidant activities

The liver and gills of *O. niloticus* (*n* = 9 for each treatment group) were removed on ice to prevent tissue squeezing. The samples were then rinsed with isotonic saline, dried and weighed. The tissues were quickly homogenized in 20% w/v ice-cold 50 mM phosphate buffer (pH 7.4) containing 1% Triton X-100 via a tissue homogenizer (Omni TH-01) at 1000 rpm for 20 s at 10 s intervals. In a cooling centrifuge, the homogenate was spun for 15 min at 4 °C at 6000 rpm. For the tissue enzyme activity assay, the supernatant was immediately collected and added to commercial test kits (Biodiagnostic, Egypt). Antioxidant activities were determined following the methods of Mohammady et al.^34^. MDA, the breakdown product of lipid peroxidation, can combine with thiobarbituric acid (TBA) to generate red products. The highest absorption peak is found at 532 nm.

### Histopathological examination

The dehydrated tissues (liver, gill, and middle intestinal portions) were placed in paraffin blocks and sliced into 5 μm sections. The sections were stained with hematoxylin and eosin (H&E)^35^. A Leica DM 500 phase-contrast microscope coupled to an ICC50W camera was used to take photomicrographs.

### Intestinal histometric examination

The histometric characteristics of the intestine, including villus length, villus width, intervilli space, and goblet cell number, were determined via Radu-Rusu et al.^36^ techniques. Using ImageJ analysis software (National Institutes of Health, MD, USA), histometric analysis was carried out, and measurements of the length, width, and intervilli space of the intestinal villi are reported in micrometers. The online application may be downloaded for free at https://imagej.nih.gov/ij/download.html.

### *P. aeruginosa* challenge test

The resistance of the fish to *P. aeruginosa* challenge was examined at the end of the trial. *P. aeruginosa* was previously isolated from diseased *O. niloticus* and molecularly identified^37^. Twenty fish/group were I/P inoculated with 0.2 ml of bacterial cell suspension containing 3 × 10^7^ cells/mL according to Shabana et al.^37^. For one week, the challenged fish were observed to document their clinical signs and mortality.

### Statistical analysis

The data obtained were subjected to the Shapiro–Wilk test and Bartlett test for evaluation of normality and homogeneity. Afterward, one-way ANOVA was used to assess the data (SPSS^®^ version 22, SPSS Inc., IL, USA). The means ± standard errors were used to express the variables. When variations were identified between treatments, the means were compared via Duncan’s post hoc test and Tukey’s test. The treatment effects were deemed significant at *P* < 0.05. The nonparametric Kruskal‒Wallis test and the Mann‒Whitney U test (*P* < 0.05) were used to compare the major changes in RBC morphology and histopathology between the groups. The changes in RBC morphology and histopathology were assessed on six-point and eight-point scales, respectively.

### Method validation and biosafety measures

Each treatment was carried out in triplicate, consisting of 3 samples from different fish per triplicate (*n* = 9 samples/treatment), to evaluate biological variation and repeatability. Quality assurance and control are essential for ensuring reliable and accurate results. For each set of assays and each time a new bottle of reagent was used, control samples, both normal and abnormal, were used. Additionally, several steps have been taken to guarantee accurate and trustworthy results. These include routine equipment maintenance and calibration, the use of positive and negative controls, and environmental monitoring to prevent false results.

As soon as the trial was over, all of the dead fish and remaining fish were destroyed in the laboratory stationary incinerator. The biosafety measures followed the pathogen regulatory guidelines for infectious materials (*P. aeruginosa*). Because PD can be highly toxic to organisms, it is necessary to control its contamination. All of the Petri dishes and equipment were cleaned with distilled water and acetone to prevent contamination during the study.

## Results

### Clinical examination and gross pathology

There were no behavioral or clinical abnormalities in the CTR or AZ groups. In the PD-exposed groups (0.5 PD and 1 PD), mild to moderate instances of gasping, convulsion, weakness, surface breathing, body imbalance, a stagnant position, and operculum movement were noted.

**Fig. 1:**
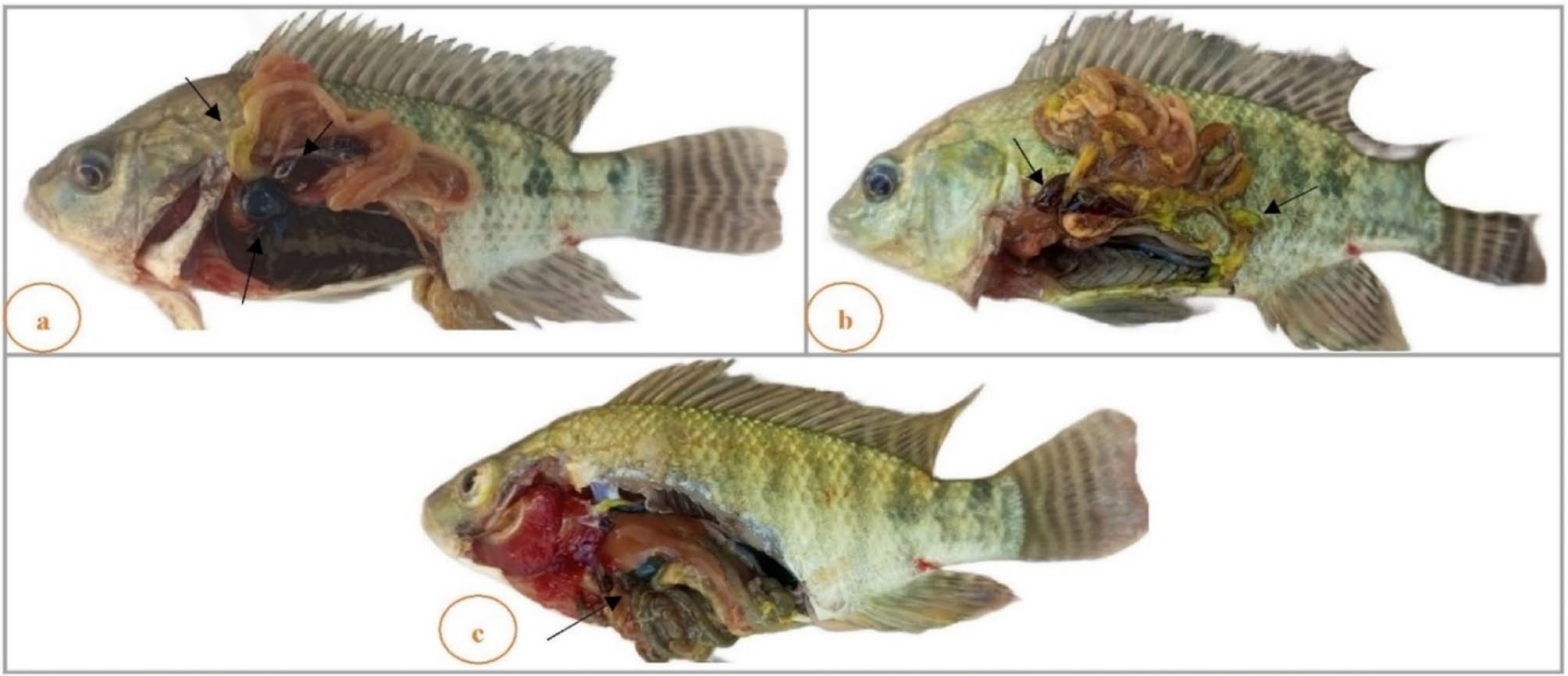
Clinical examination and gross pathology of *O. niloticus* exposed to different concentrations of PD. (**a**) 0.5 PD group showing partially yellowish-stained and empty intestines, distention of the gall bladder, and splenomegaly; (**b**) 1 PD group showing yellowish pigmentation of the skin and abdominal viscera and splenomegaly; (**c**) 1 PD + AZ group showing slight pigmentation of the skin and viscera and distention of the gall bladder.

Clinical examination of the fish revealed yellowish pigmentation of the skin (Fig. 1b, c), and P/M examination of the fish revealed splenomegaly (Fig. 1a, b) and distension of the gall bladder (Fig. 1a, c). Additionally, the gills and intestine presented yellowish pigmentation (Fig. 1a, b, c). The signs were more severe in the 1 PD group than in the 0.5 PD and PD + AZ groups.

### Growth performance

Table 4 shows the variation in growth indices among the different groups. The AZ treatment had the best performance (*P* < 0.05) in terms of W1, WG, G%, TFI, SGR, PI, and PER. This included both the AZ and 0.5 PD + AZ groups. The growth indices of the *O. niloticus* in the 1 PD group markedly decreased, except for FCR, indicating poorer performance. The 0.5 PD group did not significantly decrease most of the growth parameters. The FCR did not significantly decrease in the AZ or 0.5 PD + AZ groups. The 1 PD + AZ group presented nonsignificant increases in all growth and body indices. SR reached 100% in the CTR, AZ, and 0.5 PD + AZ treatments, whereas fish exposed to PD (0.5 and 1 mg/L PD) presented substantial decreases in the SR%. Treatment with 1 PD + AZ did not significantly decrease the SR% (95.55%). Overall, AZ treatment appeared to be the most effective at promoting growth and survival.

**Table 4:** Effects of AZ on the growth and body indices of *O. niloticus* in the experimental groups.

Parameters	CTR	AZ	0.5 PD	1 PD	0.5 PD + AZ	1 PD + AZ	*P*- value
W0 (g)	33.95 ± 0.01	34.16 ± 0.03	34.50 ± 0.35	34.21 ± 0.13	34.17 ± 0.05	34.06 ± 0.09	0.359
W1 (g)	44.72 ± 0.28^b^	49.10 ± 0.40^a^	42.14 ± 0.64^bc^	39.69 ± 0.94^c^	47.89 ± 0.11^a^	43.75 ± 0.25^b^	0.000
WG (g)	10.78 ± 0.28^b^	14.94 ± 0.37^a^	7.65 ± 0.99^bc^	5.48 ± 0.81^c^	13.72 ± 0.06^a^	9.69 ± 0.34^b^	0.005
G %	31.75 ± 0.84^b^	43.75 ± 1.04^a^	22.20 ± 3.09^bc^	16.01 ± 2.29^c^	40.17 ± 0.12^a^	28.46 ± 1.06^b^	0.005
TFI (g/fish)	30.61 ± 0.01	31.63 ± 0.03*	31.07 ± 0.29	30.20 ± 0.32	31.64 ± 0.04*	30.71 ± 0.07	0.001
FCR	2.84 ± 0.08	2.12 ± 0.05	4.14 ± 0.57^*^	5.62 ± 0.77*	2.31 ± 0.01	3.17 ± 0.12	0.009
SGR	0.43 ± 0.01^b^	0.56 ± 0.01^a^	0.31 ± 0.04^bc^	0.23 ± 0.03^c^	0.52 ± 0.001^a^	0.39 ± 0.01^b^	0.007
PI (g)	9.18 ± 0.001	9.49 ± 0.01*	9.32 ± 0.09	9.06 ± 0.10	9.49 ± 0.01*	9.21 ± 0.02	0.005
PER	1.17 ± 0.03^b^	1.57 ± 0.04^a^	0.82 ± 0.11^c^	0.60 ± 0.08^c^	1.45 ± 0.004^a^	1.05 ± 0.04^b^	0.007
SR %	100 ± 0.00	100 ± 0.00	84.44 ± 4.44*	77.78 ± 2.22*	100 ± 0.00	95.55 ± 2.22	0.000

Values (means ± SEs) with the same letters in the same row did not substantially differ (*P* < 0.05). The values in each row with an asterisk (mean ± SE) are significantly different (*P* < 0.05) from those in untreated (CTR) fish.

### Hematological and immune parameters

The Hb concentration, RBC count, and PCV of *O. niloticus* treated with PD were notably (*P* < 0.05) lower than those of the CTR group (Table 5) in a dose-dependent manner. In addition, compared with those in the CTR group, the *O. niloticus* in the PD group presented noticeable (*P* < 0.05) increases in WBC, heterophil, and monocyte counts, whereas the lymphocyte counts significantly decreased. Combined treatment with PD and AZ (125 g/kg diet) caused considerable improvements in all the hematological indices to near the CTR values. No notable differences in hematological marker levels were detected between *O. niloticus* treated with AZ alone (AZ) and those in CTR. PD toxicity caused a substantial (*P* < 0.05) decrease in all estimated immune indices (PA %, PI No, and lysosome activity) in a dose-dependent manner. Conversely, AZ markedly increased the same parameters (AZ group), and these improvements were obvious in the groups exposed to PD at both concentrations and treated with AZ (0.5 PD + AZ and 1 PD + AZ). Overall, AZ treatment was effective at promoting health and immune function in *O. niloticus*.

**Table 5:** Hematological and immune indices of *O. niloticus* exposed to different concentrations of PD and treated with AZ.

Parameters	CTR	AZ	0.5 PD	1 PD	0.5 PD + AZ	1 PD + AZ	*P*-value
Hb (g/dl)	4.67 ± 0.03^a^	4.78 ± 0.17^a^	3.36 ± 0.04^c^	2.89 ± 0.15^d^	4.41 ± 0.08^ab^	4.09 ± 0.07^b^	0.000
RBCS (x10^6^/mm^3^)	2.49 ± 0.01^ab^	2.60 ± 0.06^a^	1.68 ± 0.07^d^	1.35 ± 0.07^e^	2.28 ± 0.02^b^	2.17 ± 0.02^bc^	0.000
PCV (%)	19.51 ± 0.28^a^	19.84 ± 0.33^a^	12.71 ± 0.56^c^	10.66 ± 0.35^d^	18.18 ± 0.21^a^	15.68 ± 0.37^b^	0.000
MCV (FL)	78.49 ± 01.26	76.44 ± 0.37	75.89 ± 6.47	79.04 ± 1.51	79.71 ± 0.2	72.42 ± 1.21	0.526
MCH (Pg)	18.77 ± 0.14	18.41 ± 0.26	20.01 ± 1.04	21.52 ± 2.23	19.32 ± 0.16	18.87 ± 0.17	0.388
MCHC (%)	23.92 ± 0.21	24.09 ± 0.46	26.45 ± 0.88	27.19 ± 2.30	24.24 ± 0.14	26.06 ± 0.20	0.234
WBCS (x10^3^/mm^3^)	14.22 ± 0.55^c^	14.74 ± 0.89^c^	27.31 ± 01.23^b^	34.42 ± 0.80^a^	16.59 ± 1.33^c^	24.09 ± 1.76^b^	0.000
Heterophils (%)	20.50 ± 0.50^d^	19.50 ± 1.50^d^	37.00 ± 4.00^bc^	57.50 ± 2.50^a^	28.50 ± 1.50^c^	42.50 ± 1.50^b^	0.000
Lymphocytes (%)	72.90 ± 0.00^a^	73.25 ± 1.55^a^	52.25 ± 3.55^b^	27.35 ± 3.45^d^	62.75 ± 3.05^ab^	45.80 ± 1.10^cb^	0.000
Monocytes (%)	4.50 ± 0.50^b^	4.50 ± 0.50^b^	7.00 ± 1.00^ab^	11.00 ± 1.00^a^	5.00 ± 1.00^b^	8.50 ± 0.50^b^	0.006
Eosinophils (%)	2.00 ± 1.00	2.50 ± 0.50	3.50 ± 0.50	4.00 ± 0.00	3.50 ± 0.50	3.00 ± 0.00	0.236
Basophils (%)	0.10 ± 0.00	0.25 ± 0.05	0.25 ± 0.05	0.15 ± 0.05	0.25 ± 0.05	0.20 ± 0.10	0.409
PA %	21.48 ± 0.36^b^	25.88 ± 0.41^a^	13.72 ± 0.60^d^	10.90 ± 0.39^e^	19.94 ± 0.10^b^	17.17 ± 0.19^c^	0.000
PI No	6.04 ± 0.06^b^	6.72 ± 0.19^a^	3.68 ± 0.07^e^	2.54 ± 0.06^f^	5.07 ± 0.11^c^	4.30 ± 0.11^d^	0.000
Lysozyme (ng/ml)	8.02 ± 0.06^b^	9.20 ± 0.18^a^	3.96 ± 0.14^e^	2.14 ± 0.06^f^	6.17 ± 0.09^c^	5.14 ± 0.11^d^	0.000

The values (means ± SEs) with the same letters in the same row did not substantially differ (*P* < 0.05). (*n* = 9/group)

### Erythrocyte abnormalities

**Fig. 2:**
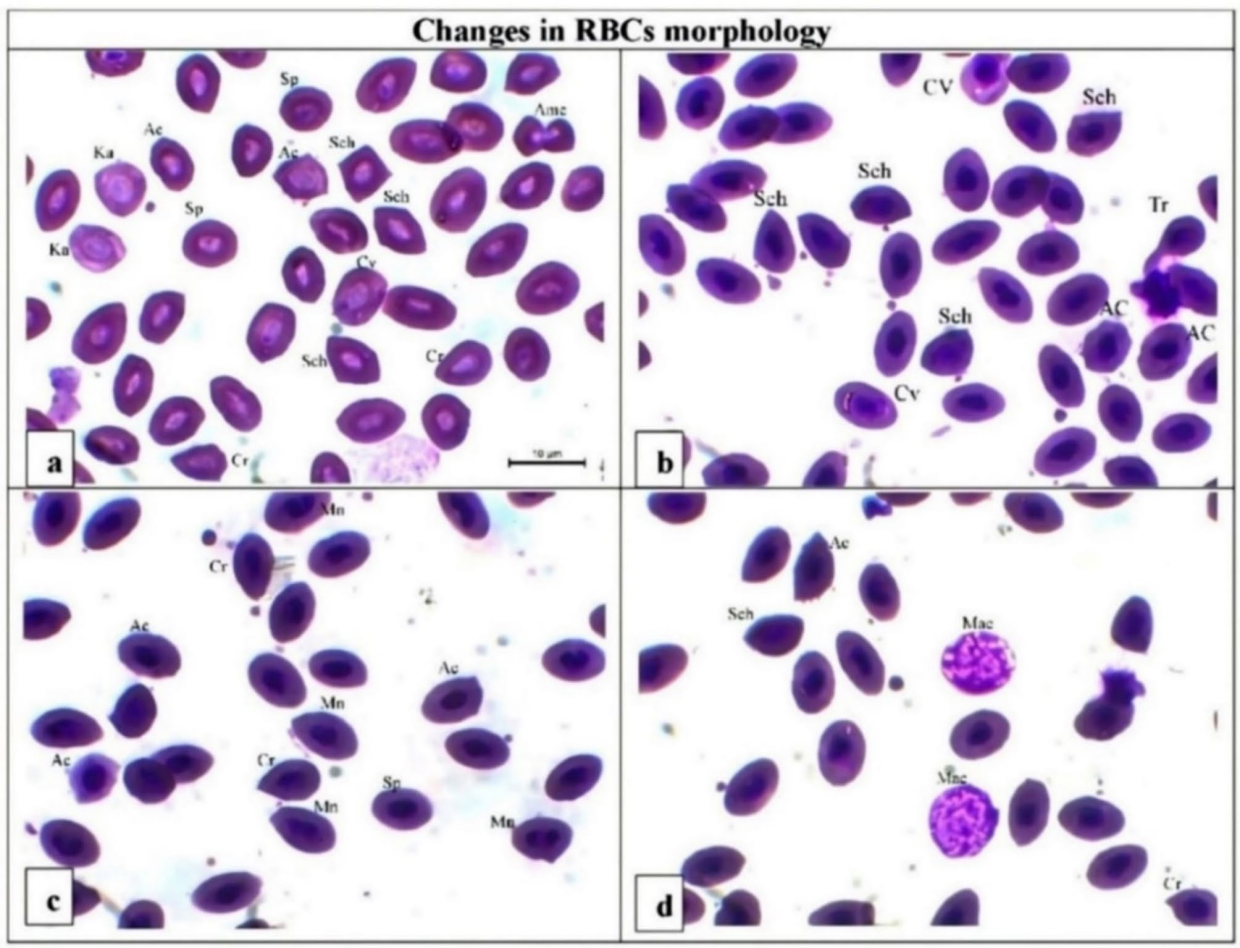
Blood films of *O. niloticus* in the experimental groups. Scale bar = 10 μm. (**a**) 1 PD group, (**b**) 1 PD + AZ group, (**c**) 0.5 PD group, (**d**) 0.5 PD + AZ group. *Ac* acanthocytes, *Sch* schinsocytes, *Cr* crenated cells, *Tr* teardrop-like cells, *AMC* amoeboid cells, *Mn* micronuclei, *Ka* karyolysis, *Sp* spherical cells, *Mac* macrophage, *Nm* nucleomegaly, *Cv* cytoplasmic vacuolation. Acanthocytes are Spiky RBCs; schinsocytes are fragmented with different shapes (triangular, comma shaped, or helmet shaped); crenated cells are cells with short, sharply pointed projections; and karyolysis is the complete dissolution of the chromatin.

The percentages of different morphological changes in the RBCs of PD-exposed fish are shown in Table 6; Fig. 2. The RBCs of the fish exposed to PD (0.5 and 1 mg/L) presented high rates of various morphological defects in a dose-dependent manner. The percentages of acanthocytes, schinsocytes, crenated cells, spherocytes and micronuclei (Fig. 2) in the fish exposed to 0.5 and 1 mg/L PD were significantly greater than those in the CTR group. Among the RBCs of the fish exposed to 0.5 and 1 mg/L PD and fed diets containing 12.5% AZ, the percentages of the different changes in RBC morphology were much lower than those of the groups exposed to PD without AZ (Table 6).

**Table 6:** Changes in RBC morphology caused by PD in different *O. niloticus* groups quantified via a six-point rating scale.

Item	CTR	AZ	0.5 PD	1 PD	0.5 PD + AZ	1 PD + AZ	*P*-value
Ac	0.44 ± 0.06^d^	0.42 ± 0.08^d^	2.43 ± 0.08^b^	4.27 ± 0.15^a^	0.48 ± 0.23^d^	1.49 ± 0.14^c^	0.021
Cr	0.27 ± 0.01^c^	0.0 ± 0.0^d^	0.75 ± 0.03^b^	1.41 ± 0.17^a^	0.29 ± 0.03^c^	1.53 ± 0.13^a^	0.032
Tr	0.0 ± 0.0^c^	0.0 ± 0.0^c^	0.68 ± 0.04^b^	0.98 ± 0.14^a^	0.21 ± 0.03^b^	0.0 ± 0.0^c^	0.000
Sch	0.36 ± 0.04^c^	0.16 ± 0.04^d^	2.52 ± 0.10^b^	3.89 ± 0.02^a^	0.39 ± 0.12^d^	0.46 ± 0.18^d^	0.042
Amc	0.0 ± 0.0	0.0 ± 0.0	0.0 ± 0.0	0.79 ± 0.05*	0.0 ± 0.0	0.0 ± 0.0	0.024
Mn	0.30 ± 0.02^d^	0.29 ± 0.07^d^	2.30 ± 0.12^b^	3.78 ± 0.12^a^	0.34 ± 0.03^d^	1.56 ± 0.98^c^	0.035
Ka	0.0 ± 0.0	0.0 ± 0.0	0.0 ± 0.0	1.23 ± 0.09*	0.0 ± 0.0	0.0 ± 0.0	0.045
Mac	0.14 ± 0.03^d^	0.42 ± 0.03^b^	0.28 ± 0.14^c^	0.23 ± 0.03^c^	0.98 ± 0.14^a^	0.05^d^ ± 0.12	0.000
Sp	0.0 ± 0.0^d^	0.0 ± 0.0^d^	0.38 ± 0.13^b^	1.78 ± 0.07^a^	0.0 ± 0.0^d^	0.18 ± 0.09^c^	0.012
Cv	0.0 ± 0.0	0.0 ± 0.0	2.04 ± 0.13*	1.81 ± 0.20*	0.0 ± 0.0	0.0 ± 0.0	0.038
Nm	0.0 ± 0.0	0.0 ± 0.0	0.82 ± 0.02*	0.0 ± 0.0	0.0 ± 0.0	0.0 ± 0.0	0.048

*Ac* acanthocytes, *Sch* schinsocytes, *Cr* crenated cells, *Tr* teardrop-like cells, *AMC* amoeboid cells, *Mn* micronuclei, *Ka* karyolysis, *Sp* spherical cells, *Mac* macrophage, *Nm* nucleomegaly, *Cv* cytoplasmic vacuolation.

For each group, the values are the average of nine observations (*n* = 9/group). The values (means ± SEs) with the same letters in the same row did not substantially differ (*P* < 0.05). The values in each row with an asterisk (mean ± SE) are significantly different (*P* < 0.05) from those in untreated (CTR) fish.

*Morphological alterations ordinal scale: 0 = None; 1 = normal with less than 5% of RBCs affected; 2 = mild with 5–15% of RBCs affected; 3 = moderate with 15–25% of RBCs affected; 4 = marked with 25–50% of RBCs affected; and 5 = severe with more than 50% of RBCs affected.

### Biochemical parameters

Dose-dependent increases in the levels of hepatic damage indicators (AST, ALT, and ALP) and renal damage indicators (urea and creatinine) were detected in *O. niloticus* in the PD groups compared with those in the CTR group (Table 7). The serum cortisol and glucose levels of the fish in the PD groups, especially those in the 1 PD group, also exhibited considerable increases (*P* < 0.05). In contrast, a significant decrease in ACHE was detected in the PD-exposed groups compared with the CTR group. Once again, the addition of AZ to the diet of PD-exposed fish restored all the aforementioned parameters to levels closer to those of the CTR group. The CTR group and AZ group had almost the same levels of the measured biochemical indices. Total protein and globulin were notably augmented in the AZ-supplemented group.

**Table 7:** Stress and biochemical biomarkers of *O. niloticus* exposed to different concentrations of PD and treated with AZ.

Parameter	CTR	AZ	0.5 PD	1 PD	0.5 PD + AZ	1 PD + AZ	*P*-value
Glucose (mg/dL)	43.33 ± 3.18^bc^	56.00 ± 2.51^b^	56.00 ± 2.00^b^	72.33 ± 4.26^a^	35.33 ± 1.77^c^	34.00 ± 2.08^c^	0.000
Cortisol (ng/mL)	5.13 ± 0.15^c^	5.61 ± 1.18^c^	7.23 ± 0.38^b^	10.69 ± 0.37^a^	4.70 ± 0.28^c^	6.86 ± 0.29^bc^	0.000
ACHE (U/L)	440.47 ± 4.89^a^	450.13 ± 1.16^a^	386.80 ± 2.10^c^	316.96 ± 3.03^d^	449.20 ± 0.81^a^	415.88 ± 1.79^b^	0.000
TP (g/dL)	2.99 ± 0.22	4.66 ± 0. 66*	2.34 ± 0.08	2.18 ± 0.18	2.99 ± 0.06	2.57 ± 0.37	0.002
Albumin (g/dL)	1.57 ± 0.13	1.61 ± 0.18	1.53 ± 0.08	1.32 ± 0.03	1.50 ± 0.09	1.75 ± 0.08	0.202
Globulin (g/dL)	1.42 ± 0.32^b^	3.06 ± 0.68^a^	0.81 ± 0.13^b^	0.86 ± 0.19^b^	1.50 ± 0.02^ab^	0.82 ± 0.37^b^	0.005
A/G ratio	1.27 ± 0.39^ab^	0.58 ± 0.14^b^	1.10 ± 0.37^ab^	1.68 ± 0.37^ab^	1.00 ± 0.07^ab^	2.98 ± 0.94^a^	0.045
ALT (IU/L)	30.91 ± 1.53^c^	32.72 ± 3.12^bc^	42.44 ± 2.47^b^	57.69 ± 2.48^a^	25.92 ± 1.47^c^	24.03 ± 1.53^c^	0.000
AST (IU/L)	51.14 ± 1.54^bc^	45.04 ± 2.05^c^	60.29 ± 1.81^b^	76.58 ± .55^a^	49.00 ± 2.31^bc^	51.92 ± 4.47^bc^	0.000
ALP (IU/L)	14.80 ± 1.16^c^	14.33 ± 5.09^c^	22.73 ± 7.86^b^	41.70 ± 2.97^a^	10.16 ± 1.65^c^	23.54 ± 2.74^b^	0.003
Urea (mg/dL)	14.02 ± 0.67^b^	11.05 ± 0.57^b^	22.37 ± 1.22^a^	29.72 ± 3.50^a^	11.14 ± .60^b^	18.13 ± 0.60^ab^	0.000
Creatinine (mg/dL)	0.37 ± 0.02^bc^	0.26 ± 0.03^c^	0.93 ± 0.06^a^	1.17 ± 0.02^a^	0.35 ± 0.03^bc^	0.60 ± 0.14^b^	0.000

The values (means ± SEs) with the same letters in the same row did not substantially differ (*P* < 0.05). The values in each row with an asterisk (mean ± SE) are significantly different (*P* < 0.05) from those in untreated (CTR) fish. (*n* = 9/group)

### Tissue antioxidant capacity and serum electrolyte balance

**Fig. 3:**
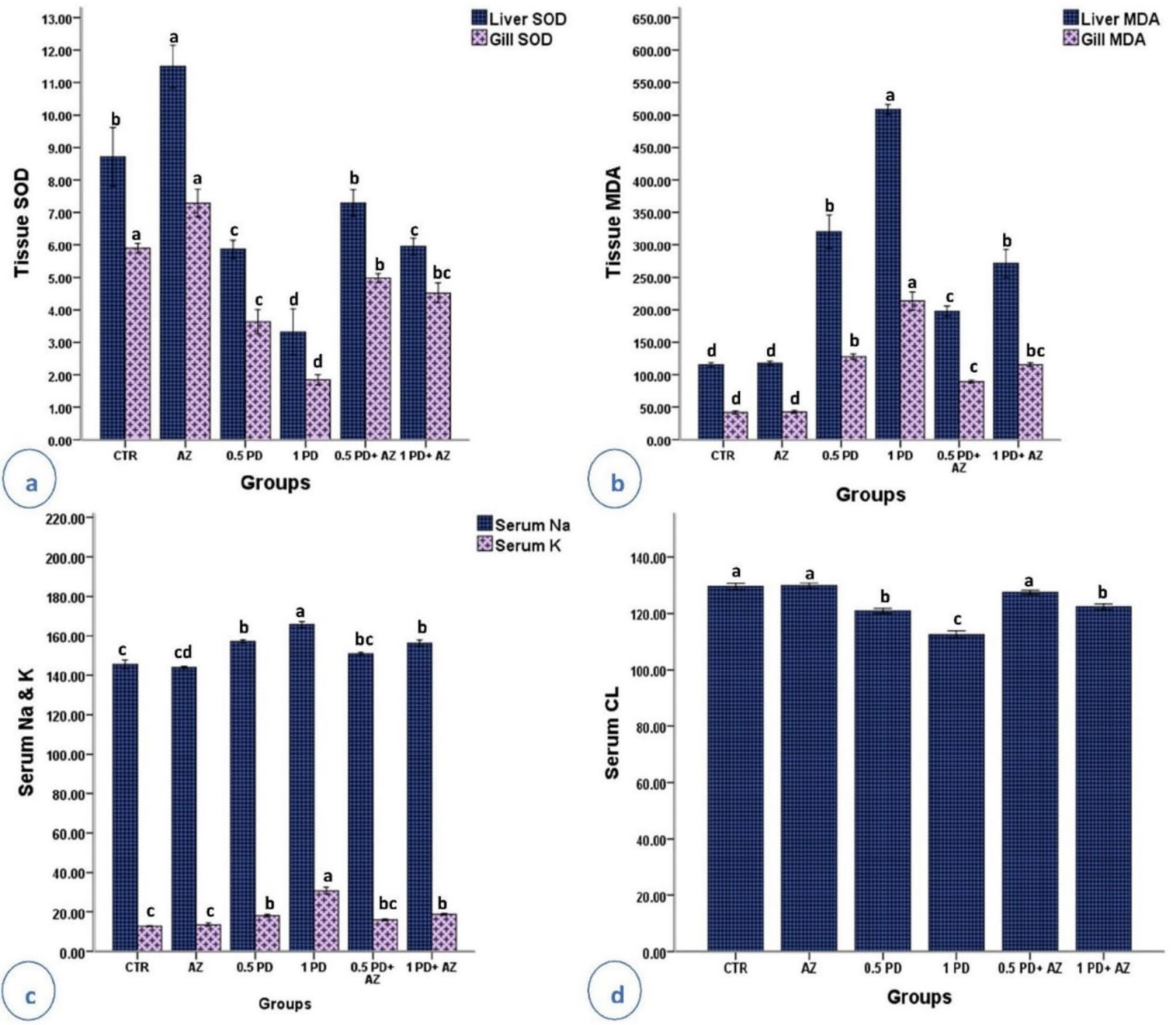
Tissue antioxidant capacity and serum electrolytes of *O. niloticus* in the experimental groups. (**a**) Hepatic and gill SOD: superoxide dismutase activity (U/g); (**b**) hepatic and gill MDA: malondialdehyde (nmol/g); (**c**) serum Na^+^: sodium and K^+^: potassium (mmol/L); (**d**) serum Cl^−^: chloride (mEq/L). The values are the means (*n* = 9/group), and the SEs are indicated by the error bars. The different subscripts above the bars indicate significant differences between treatments (*P* < 0.05).

Compared with those in the CTR group, the PD groups presented noticeable (*P* < 0.05) decreases in SOD activity in the liver and gills (Fig. 3a) and a notable (*P* < 0.05) increase in the MDA content in the same tissues in a dose-dependent manner (Fig. 3b). AZ supplementation (AZ group) resulted in the greatest increase in hepatic SOD activity, with no significant differences in gill SOD or MDA levels in either tissue (liver or gills). The oxidative stress indicators in the liver and gills improved when the fish were exposed to PD and treated with AZ.

The effects of PD on the serum electrolyte balance of *O. niloticus* are presented in Fig. 3c and d. K + activity increased in a dose-dependent manner, whereas the serum Cl- level was significantly lower in the PD group. Fish exposed to PD at two different concentrations (0.5 and 1 mg/L) presented a dose-dependent increase in the serum Na^+^ concentration. Adding AZ to the diets of PD-treated fish significantly improved the serum electrolyte balance toward CTR values. Overall, AZ treatment showed a protective effect, restoring SOD and MDA levels and the serum electrolyte balance closer to those of the CTR group, mitigating the negative effects of PD exposure.

### Histological evaluation

**Fig. 4:**
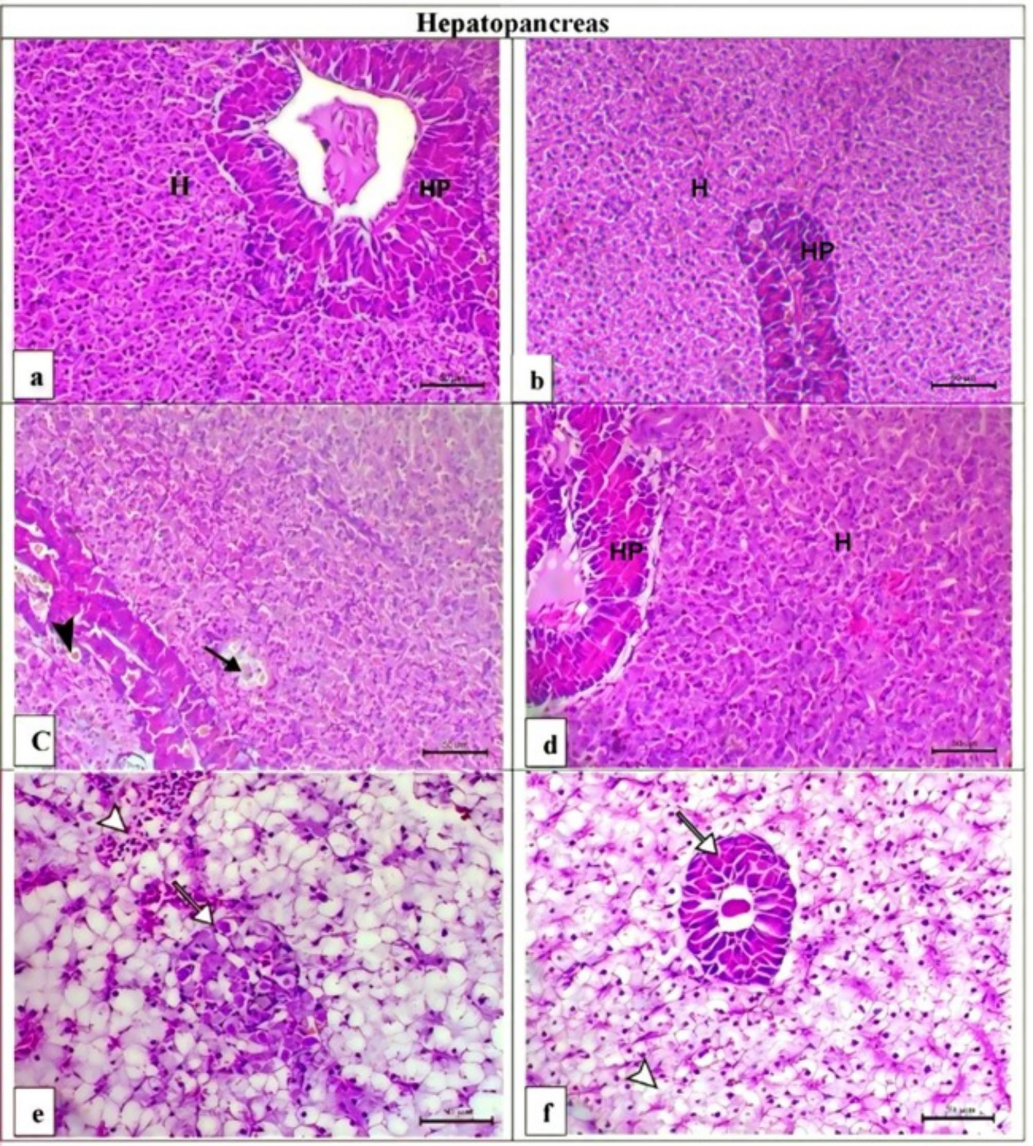
Representative photomicrographs of H&E-stained hepatopancreases and gills from *O. niloticus* in the experimental groups. H&E, Scale bar = 50 μm. (**a**) The CTR group presented a normal hepatopancreatic histoarchitecture in which hepatocyte cords (H) and pancreatic acini surround the central viens (HP). (**b**) The AZ group presented normal hepatic (H) and pancreatic (HP) portions. (**c**) The 0.5 PD group showed hepatic necrosis (arrow) and diffuse activation of melanomacrophage centers (MMCs, arrowhead). (**d**) The 0.5 PD + AZ group presented normal hepatopancreatic histoarchitecture. (**e**) The 1 PD group presented severe congestion of the hepatic capillaries (arrowhead), marked vacuolar degenerative changes and necrotic changes within the pancreatic portion (arrow). (**f**) The 1 PD + AZ group presented mild hepatic vacuolation (arrowhead) and a normal hepatopancreas (arrow).

Histopathological changes in different fish tissues exposed to different dosages of PD are briefly tabulated in Table 8. The results of hepatopancreas histopathology revealed various microscopic lesions, such as hepatic necrosis, pancreatic degeneration and necrosis, vacuolar degeneration, congestion of hepatic capillaries, and an increase in the number of melanomacrophages (Fig. 4c and e), in PD-treated fish. The disrupted hepatopancreas structure was restored in the 0.5 PD + AZ (Fig. 4d) and 1 PD + AZ (Fig. 4f) groups. The hepatopancreatic structure of the CTR group (Fig. 4a) and AZ group (Fig. 4b) was normal, with hepatocyte cords and pancreatic acini surrounding the central vein.

**Table 8:** Histological alterations (evaluated quantitatively on an eight-point scale) and histomorphometry of the middle intestinal portions of *O. niloticus* exposed to different concentrations of PD and treated with AZ.

Organ	CTR	AZ	0.5 PD	1 PD	0.5 PD + AZ	1 PD + AZ	
Hepatopancreas							
HN	–	–	–	+++	–	–	
PD	–	–	–	+++	–	–	
PN				+++			
MMCs	–	–	–	–	–	–	
CHC	–	–	–	++++	–	–	
VD	–	–	–	+++	–	+	
Gills							
EN	–	–	+	+++	–	–	
MFL	–	–	^−^	+++	–	–	
EH	–	–	–	–	–	+	
LA	–	–	+++	–	–	–	
ICI	–	–	+++	–	–		
LT	–	–	–	–	+	–	
Middle intestinal portion							
VL	–	+++	––	–––	–	–	
BV	–	–	++	++++	–	–	
DC	–	–	++	+++	–	–	
MN	–	–	–	+++	–	–	
VD	–	–	+	++	–	–	
IVS	–	–	–	++	–	–	
GCN	–	++	––––	––––	––	–––	

							*P*-value
Intestinal histomorphometry							
Villi length (µm)	432.41 ± 4.88^b^	545.81 ± 2.60^a^	310.94 ± 41.29^c^	211.58 ± 19.44^d^	436.41 ± 8.33^b^	405.75 ± 6.25^b^	0.000
Villi width (µm)	77.13 ± 2.54^b^	77.32 ± 3.05^b^	107.93 ± 7.77^ab^	157.61 ± 23.17^a^	89.88 ± 8.84^b^	70.15 ± 1.39^b^	0.001
Inter villi space (µm)	65.79 ± 10.83^b^	44.00 ± 7.10^b^	81.94 ± 22.56^b^	153.03 ± 11.06^a^	63.62 ± 17.22^b^	60.28 ± 11.20^b^	0.002
Goblet cell no (no/mm^2^)	75.33 ± 3.18^b^	96.33 ± 4.67^a^	33.33 ± 3.76^d^	19.33 ± 2.33^e^	66.33 ± 2.33^bc^	57.67 ± 1.45^c^	0.000

Except for the CTR group, which had two observations for each organ, the data are the average of nine observations for each organ in the appropriate group.

*HN* hepatic necrosis, *PN* pancreatic necrosis, *PD* pancreatic degeneration, *MMCs* melanomacrophage centers, *CHC* congestion of the hepatic capillaries, *VD* vacuolar degeneration, *EN* epithelial necrosis, *MFL* multifocal lifting, *EH* epithelial hyperplasia, *LA* Lamelar adhesion, *ICI* inflammatory cell infiltration, *LT* Lamelar thickening, *VL* villus length, *BV* blunted villi, *DC* degenerative changes, *MN* mucosal necrosis, *VD* villus width, *IVS* intervilli space, *GCN* goblet cell number.

*Eight-point scale: –: no change, ++++: severe, +++: marked, ++: moderate, +: mild, ––: mild decrease, –––: decrease, and ––––: marked decrease. For intestinal histomorphometry, the values (means ± SEs) with the same letters in the same row did not substantially differ (*P* < 0.05)

**Fig. 5:**
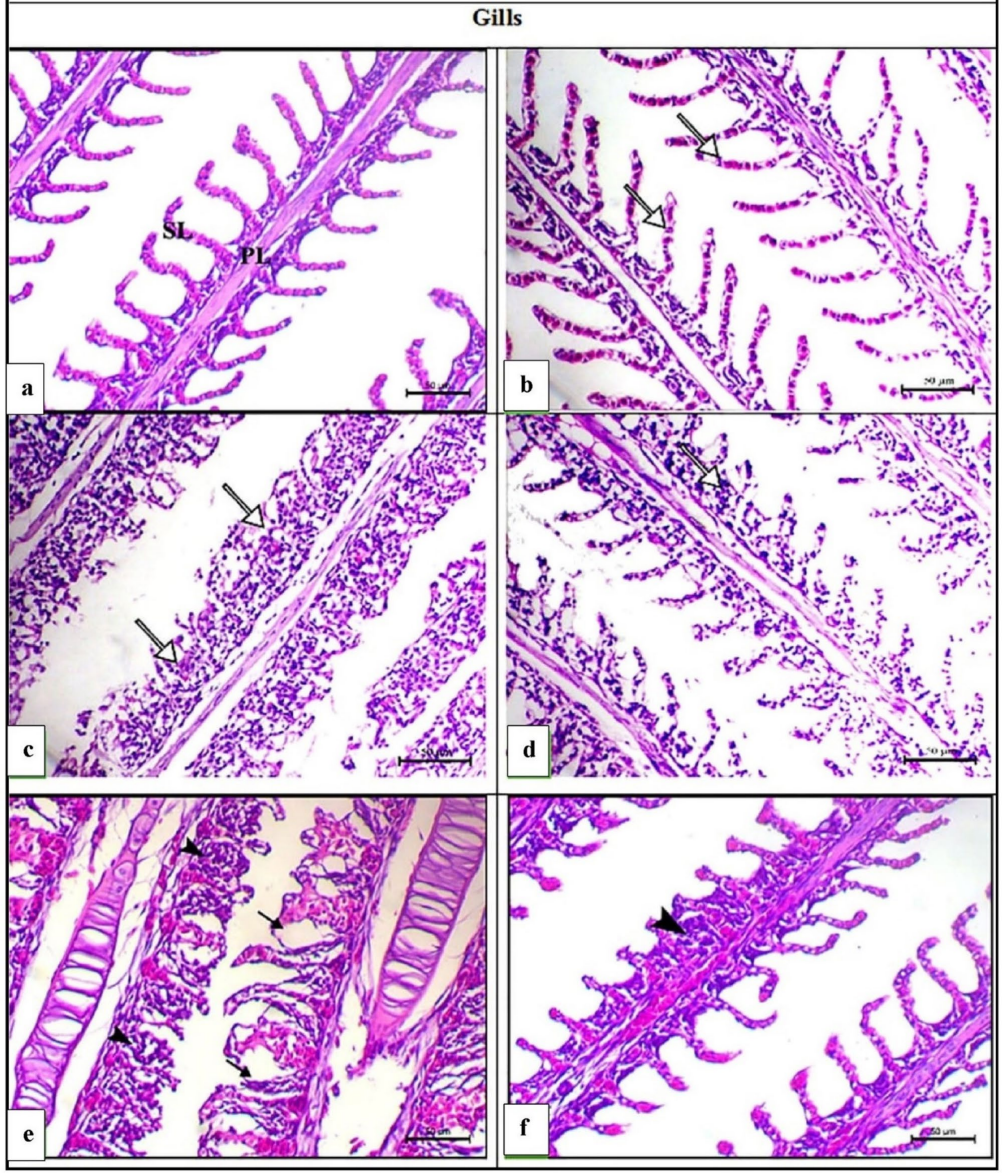
Representative photomicrographs of H&E-stained gills from *O. niloticus* in the experimental groups. H&E, Scale bar = 50 μm. (**a**) The CTR group presented a normal histoarchitecture consisting mainly of primary lamellae (PL) branching out into tiny secondary lamellae (SLs). (**b**) The AZ group showed normal secondary lamellae (arrows). (**c**) The 0.5 PD group showed marked adhesion of the secondary lamellae associated with marked inflammatory cell infiltration (arrows). (**d**) The 0.5 PD + AZ mild thickening of the secondary lamellae (arrow). (**e**) The 1 PD group presented diffuse covering epithelial necrosis of primary lamellae (arrowheads) and multifocal lifting of secondary lamellae (arrows). (**f**) The 1 PD + AZ group presented focal epithelial hyperplasia of some primary lamellae (arrowhead).

Severe histopathological changes, such as epithelial necrosis, multifocal lifting, epithelial hyperplasia, thickening and adhesion, and inflammatory cell infiltration, were detected in the gills of the fish at 0.5 PD (Fig. 5c) and 1 PD (Fig. 5e). Dietary inclusion of AZ significantly restored the disturbed gill structure in the 0.5 PD + AZ (Fig. 5d) and 1 PD + AZ (Fig. 5f) groups. The CTR group (Fig. 5a) and AZ group (Fig. 5b) presented a normal histoarchitecture consisting mainly of primary lamellae branching out into tiny secondery lamellae.

**Fig. 6:**
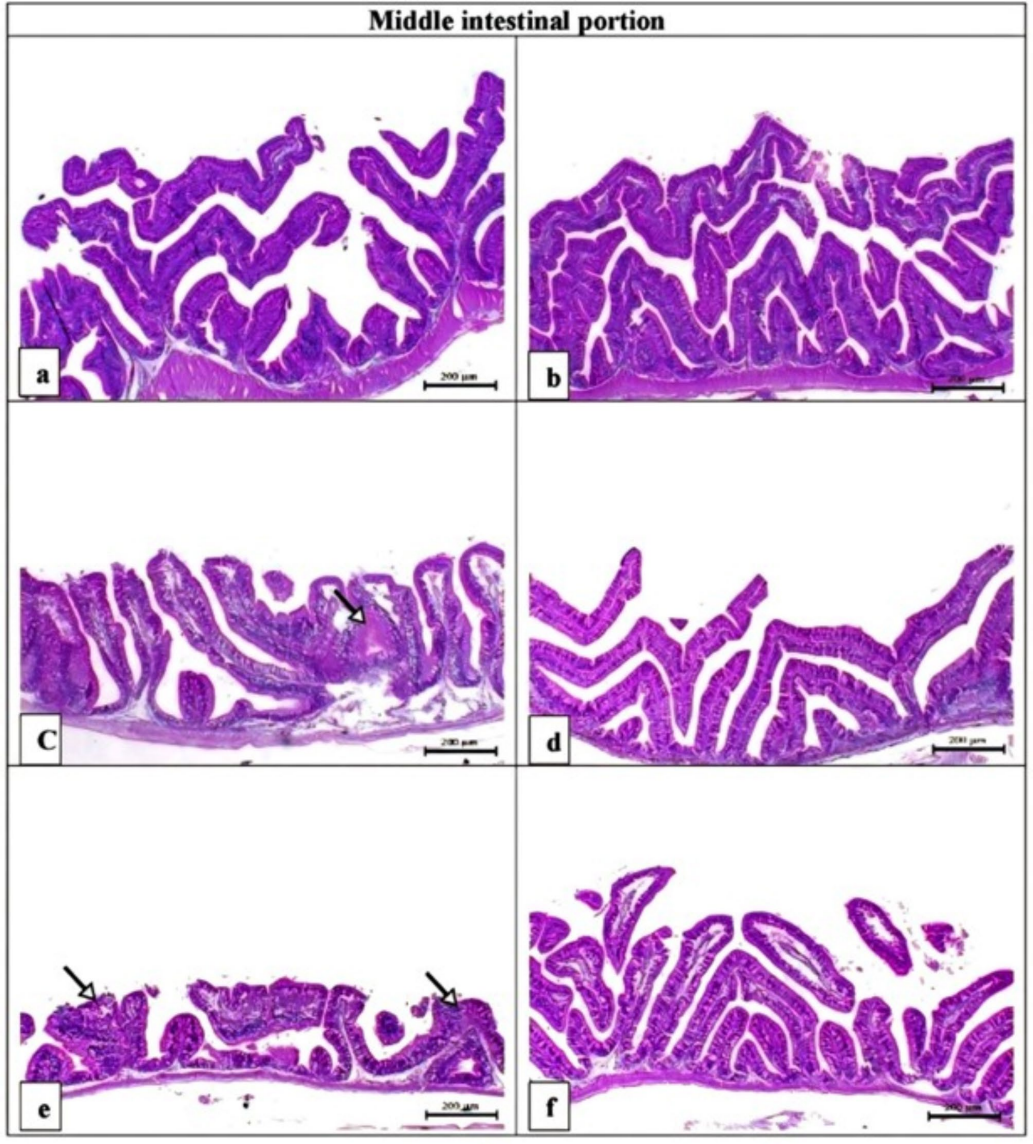
Representative photomicrograph of the H&E-stained middle intestinal portions of *O. niloticus* in the experimental groups. Scale bar = 200 μm. (**a**) The CTR group presented normal intestinal villus length and branches. (**b**) The AZ group presented a marked increase in intestinal villus length and branch number. (**c**) The 0.5 PD group presented blunted intestinal villi with degenerative changes in the mucosal lining (arrow). (**d**) The 0.5 PD + AZ group presented normal intestinal villus length. (**e**) The 1 PD group presented marked blunting of the intestinal villi associated with marked degeneration and necrosis of the intestinal mucosa (arrows). (**f**) The 1 PD + AZ group presented a normal intestinal villus length.

Histopathological examination and histometric measurements of the middle intestinal portions of *O. niloticus* are presented in Fig. 6; Table 8. In the middle intestinal portion of the PD-treated groups (6c and e), moderate to marked histopathological alterations, such as blunted villi, degenerative changes, mucosal necrosis, decreased villus length, increased villus width, increased intervilli space, and decreased goblet cell number, were observed. AZ increased villus length and the number of goblet cells (Fig. 6b) and even restored the disrupted intestinal structure induced by PD in the 0.5 PD + AZ and 1 PD + AZ groups (Fig. 6d, f). Major histopathological alterations were detected in the tissues (hepatopancreas, gills, and middle intestinal portion) of the fish in the 1 PD group.

### Resistance of the fish to *P. aeruginosa*

**Fig. 7:**
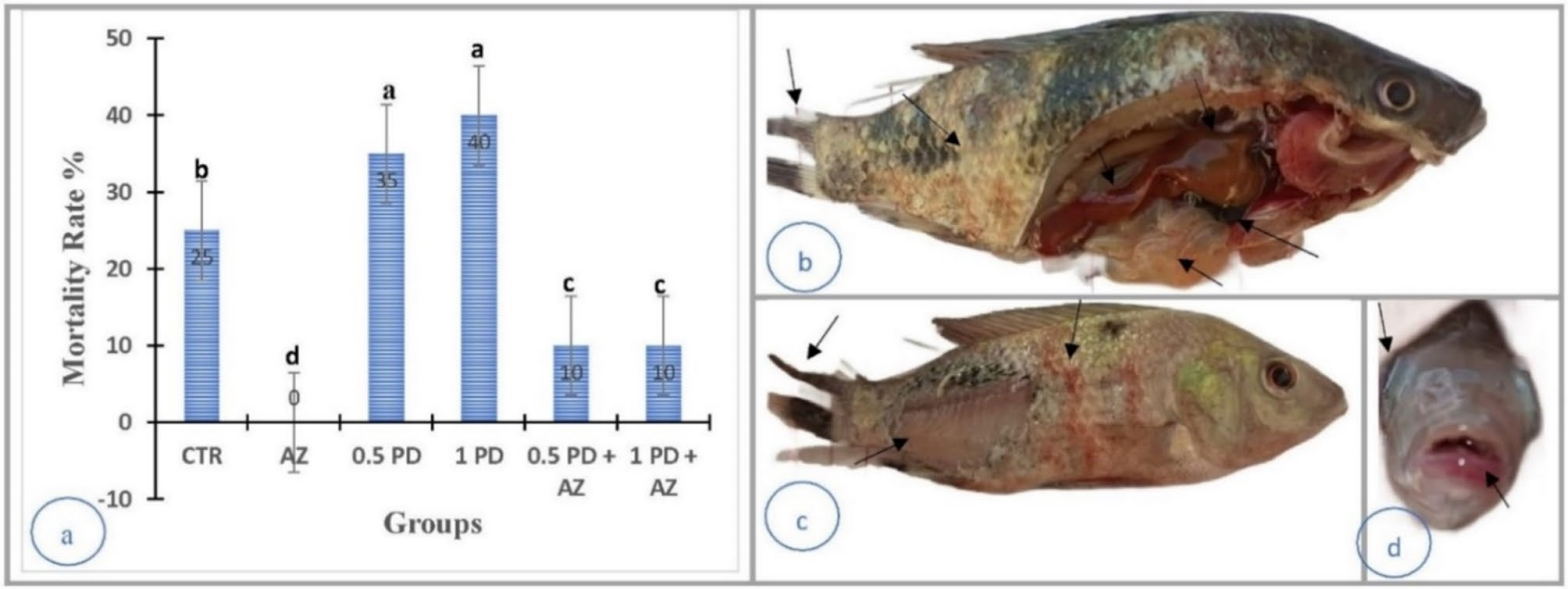
Resistance of fish to *P. aeruginosa* infection. (**a**) Mortality rate. (**b–d**) Clinical examination and gross pathology of *O. niloticus* in the experimental groups after bacterial challenge. (**b**) 1 PD group showing skin pigmentation, an eroded tail, a hemorrhagic liver, retention of bile in the liver, distension of the gallbladder, and an empty intestine; (**c**) 0.5 PD group showing skin pigmentation, ulcers, and hemorrhage and an eroded tail; (**d**) 0.5 PD group showing ulcers in the oral region and eye protrusion.

The challenge presented by bacterial agents such as *P. aeruginosa* is a highly effective assay for measuring the active immune responses of fish. The group supplemented with AZ presented no mortality, whereas the CTR group presented 25% mortality (Fig. 7a). The mortality in the 0.5 PD and 1 PD groups after the challenge reached 35% and 40%, respectively, whereas these values decreased to 10% upon AZ supplementation in both the 0.5 PD + AZ and 1 PD + AZ groups.

The fish in the PD-exposed groups presented changes in skin pigmentation (Fig. 7b, c), which can be indicative of stress or infection. Tail erosion (Fig. 7b, c) is a common sign of bacterial infection and can impair a fish’s ability to swim effectively. Skin ulcers and hemorrhages were observed in the PD-exposed groups (Fig. 7c). These lesions are indicative of severe bacterial infection and compromised skin integrity. The fish in the CTR group presented ulcers in the oral region and eye protrusions (Fig. 7d). Oral ulcers can affect feeding behavior, whereas eye protrusions (exophthalmia) can be associated with systemic infection. Postmortem (P/M), the 1PD group (Fig. 7b) presented a hemorrhagic liver and retention of bile in the liver. These signs indicate hepatic damage and potential disruption of liver function. Distension of the gallbladder was observed in the PD group. This can be indicative of bile retention and impaired bile excretion, which affect digestion and metabolism. The 1 PD group also presented an empty intestine. This may suggest reduced feeding activity or digestive dysfunction due to infection. The fish in the combined treatment groups presented fewer signs of infection, indicating the potential efficacy of the treatment in mitigating the impact of *P. aeruginosa*. The AZ group appeared to be in better health during the challenge, exhibiting fewer clinical signs of infection, suggesting a protective effect of the treatment.

## Discussion

In this study, we examined the effects of dietary AZ supplementation on the growth, survival, hematological parameters, immune response, erythrocyte morphology, and tissue histology of *Oreochromis niloticus* exposed to varying concentrations of PD. Our results significantly contribute to the understanding of the potential benefits of AZ in aquaculture and the mechanisms underlying these effects.

The observed behavioral abnormalities in the PD-exposed groups, including gasping, convulsion, weakness, surface breathing, body imbalance, a stagnant position, and operculum movement, indicate a stress response. Stress can be due to disruption of homeostasis and interference with normal metabolic processes, leading to energy depletion and physiological imbalance. These findings are supported by our histological findings, which revealed gill damage, leading to operculum movement and surface breathing^38^.

The decrease in growth indices such as WG, SGR, and PI in the 1 PD group is likely due to the negative impact of PD on metabolic and physiological processes. Herbicides can interfere with protein synthesis, nutrient absorption, and energy metabolism, leading to reduced growth performance^39^. The critical effects of PD, including liver toxicity, intestinal histological damage, and hematological and biochemical changes, likely contributed to the decreased body weight observed in our study. However, the addition of 12.5% AZ to the diet substantially increased all growth parameters, which is consistent with the findings of Magouz et al.^14^, demonstrating the positive impact of AZ on growth performance. The improvement in growth indices in the AZ-supplemented groups suggested that AZ mitigated these adverse effects, potentially by providing essential nutrients and increasing metabolic efficiency^14^.

Survival rates were also adversely affected by PD exposure, with decreased survival observed at both 0.5 PD and 1 PD concentrations. This trend is corroborated by previous studies on *O. niloticus* exposed to PD^6^. Conversely, AZ supplementation improved survival rates, particularly in the PD-treated groups, indicating the protective role of AZ in mitigating PD toxicity.

PD exposure leads to anemia, as evidenced by a lower Hb concentration, RBC count, and PCV. Anemia can result from hemolysis or impaired erythropoiesis due to oxidative stress and damage to hematopoietic tissues^40,41^. The increases in WBCs, heterophils, and monocytes indicate an immune response to infection or inflammation^41^. AZ supplementation appears to restore these indices, suggesting its role in supporting hematopoiesis and immune function. The phenolic compounds in AZ may play a role in protecting erythrocyte membranes from PD-induced damage^42^. Additionally, the improved immune response, as evidenced by increased serum lysozyme levels, total serum protein, and phagocytic activity, highlights the immunomodulatory and antistress effects of AZ. Interestingly, coadministration of AZ and PD restored the hematological and immune parameters to the CTR values, confirming the immunomodulatory and antistress effects of AZ. This might be the result of the antioxidant properties of AZ triggering its defense mechanisms.

The high rates of acanthocytes, schistocytes, crenated cells, spherocytes, and micronuclei suggest oxidative damage to RBCs and impaired erythropoiesis^43^. These changes can result from the generation of reactive oxygen species (ROS) due to PD exposure^38^. These alterations are consistent with previous studies on fish exposed to various environmental contaminants^44,45^. The increased membrane permeability and lipid peroxidation induced by PD likely contribute to these morphological changes. AZ appears to reduce these morphological defects, likely due to its antioxidant properties^46^.

Elevated levels of AST, ALT, ALP, urea, and creatinine indicate hepatic and renal damage caused by PD. PD exposure can induce hepatotoxicity, nephrotoxicity, and impaired protein breakdown through oxidative stress and inflammation^12^. Additionally, high levels of urea and creatinine indicate that the kidney is unable to fully remove these byproducts from the blood, which may be the result of cellular damage following pesticide exposure^12,47^. Azolla’s ability to reduce these levels suggests its protective role against organ damage through antioxidant mechanisms that inhibit lipid peroxidation and preserve membrane integrity.

Elevated blood levels of cortisol and glucose in PD-exposed fish reflect a stress-induced gluconeogenesis response in an effort to meet their energy requirements, which is consistent with studies by El-Sharkawy et al.^39^ and Mrong et al.^41^. Furthermore, any toxicant-induced hyperglycemia may be explained by the suppression of the adrenal medulla’s neuroeffector sites, which results in hypersecretion of adrenaline and promotes the conversion of glycogen to glucose^39^. The concurrent administration of AZ reduced cortisol and glucose levels, enhancing the ability of fish to combat infections through improved local immunity^48^. The antistress effects of AZ could be attributed to its anti-inflammatory properties and antioxidant activity. Studies indicate that oxidative stress may be connected to pesticide-induced AChE activity reduction^3,40^. The inhibition of AChE activity affects essential physiological processes such as predator escape and food movement. AZ administration notably increased AChE levels, restoring them toward CTR values and thereby mitigating the toxic effects of PD.

PD exposure leads to oxidative stress, as indicated by reduced SOD activity and increased MDA levels. This reduction in SOD activity is critical, as SOD is one of the primary defenses against reactive oxygen species (ROS). Depletion of hepatic antioxidant enzymes, possibly due to oxidative damage generated during the detoxification of contaminants^49^, was observed. Elevated malondialdehyde (MDA) levels in PD-exposed fish suggest the occurrence of ROS-induced disruption of cellular membranes^50^. AZ supplementation enhances antioxidant defense mechanisms, reducing oxidative stress. The antioxidant properties of AZ, which involves binding metal ions, replenishing membrane-bound antioxidants, and decreasing ROS, serve as the main defense mechanism against PD toxicity^51^.

The results of the electrolyte analysis revealed that the ability of fish exposed to PD to sustain gaseous exchange, osmotic equilibrium, and nerve impulse contraction was impaired. Significant dose-dependent variations in serum electrolytes were observed, with notable decreases in Cl- levels and increases in Na + and K + levels in PD-exposed fish. This imbalance indicates that intoxicated fish are unable to maintain their integrity through their gills, resulting in an unbalanced state within their environment. The observed electrolyte imbalance may stem from gill and liver damage, as both organs play vital roles in ion transport and osmoregulation. Histological evaluations revealed that PD toxicity affects the liver and gills, which are crucial for maintaining Na + and K + levels. However, dietary AZ significantly improved the serum electrolyte balance in PD-exposed *O. niloticus*, demonstrating its protective effects.

PD exposure resulted in significant microscopic lesions, such as hepatic necrosis, pancreatic degeneration and necrosis, vacuolar degeneration, congestion of hepatic capillaries, and an increase in the number of MM centers in the hepatopancreases of the fish. These findings are consistent with those reported by El-Sharkawy et al.^39^ and Elbahnaswy et al.^7^, who reported similar histological changes in *O. niloticus* exposed to PD. These changes likely result from the enterohepatic pathway of PD, as indicated by elevated serum liver enzymes and decreased total serum protein levels. The gills are critical organs for gas exchange, ion regulation, and osmoregulation. PD exposure caused severe pathological changes in the gills, including epithelial necrosis, multifocal lifting, epithelial hyperplasia, thickening and adhesion, and inflammatory cell infiltration. Similar changes were reported in *Hypophthalmichthys nobilis* exposed to sublethal concentrations of PD^52^. Once again, AZ supplementation significantly restored the disturbed liver and gill structures, demonstrating its protective effects.

Intestinal morphometric measurements, such as villus length, villus width, and goblet cell number, are crucial for understanding the mechanisms of absorption and digestion^14^. PD-treated fish presented moderate to marked histopathological alterations, including blunted villi, degenerative changes, mucosal necrosis, decreased villus length, increased villus width, increased intervilli space, and decreased goblet cell number. These changes impair the digestion and absorption of essential nutrients, adversely affecting fish growth. Conversely, AZ improved the villus length and goblet cell number, restoring the disrupted intestinal structure, which aligns with the findings of Magouz et al.^14^. AZs contain significant amounts of protein and essential amino acids that may increase nutrient absorption and promote fish growth^14^.

Our histopathological observations support the positive effects of AZ in mitigating PD-induced damage in various tissues. Fish subjected to PD poisoning presented noticeable pathological alterations in their intestines, gills, and liver, indicating a compromised immune system and tissue resistance. However, the fish that were administered AZ alone or in conjunction with PD presented minimal to no pathological alterations, suggesting improved immune function and tissue regeneration. The increase in the number of goblet cells in the intestinal mucosa of the AZ-treated groups indicates enhanced local immunological resistance against toxic effects, which is correlated with higher survival rates than those of the PD-exposed groups.

The challenge test with *P. aeruginosa* revealed greater mortality in the PD-exposed groups than in the CTR group, suggesting the presence of compromised active immune system components. The immunostimulant potential of AZ was demonstrated by significant improvements in immune response variables and increased resistance to *P. aeruginosa*, as evidenced by decreased mortality. This finding strongly correlates with increased immune parameters and SOD activity, along with decreased MDA levels, demonstrating the ROS-scavenging, antioxidant, and immunomodulatory effects of AZ. The protective effect of AZ was verified by Verma et al.^53^, who reported that adding 2.5% AZ powder could prevent disease caused by *A. hydrophila* in *Clarias gariepinus* and improve fish health by enhancing the immune response. Furthermore, the cellulose and lignin contents of AZ have positive antibacterial effects and function as prebiotics^54^. Tannins (secondary metabolites of AZ) have several biological properties, such as antibacterial, antiviral, antiparasitic, antioxidant, immunomodulatory, and anti-inflammatory effects^55^.

## Conclusion

The findings of this study have significant implications for the field of aquaculture. These results highlight the detrimental effects of PD on the health and immune response of *O. niloticus*, indicating that PD exposure can severely compromise fish health. These findings underscore the importance of regulating pesticide use in agricultural areas near fish farms to prevent contamination and protect aquatic life. Furthermore, this study demonstrated the potential of AZ as a natural, sustainable dietary supplement that can mitigate the adverse effects of PD exposure. The antioxidant and immunostimulant properties of AZ not only enhance fish growth and health but also offer a cost-effective alternative to traditional protein sources such as fish meal. These insights pave the way for the development of eco-friendly aquaculture practices that prioritize fish welfare and environmental sustainability. Future research should focus on elucidating the mechanisms underlying the immunostimulant and antioxidative effects of AZ to optimize its application in sustainable aquaculture practices.

## Limitations of the study

While the study provides valuable insights, several limitations should be acknowledged. This study was conducted under controlled laboratory conditions, which may not fully replicate the complexities of natural aquatic environments. The effects of PD and AZ could vary under field conditions due to factors such as water quality, temperature fluctuations, and interactions with other environmental contaminants. Additionally, the present study focused on a specific concentration and duration of PD exposure, and different levels or extended exposure periods may yield varying results. The dietary concentration of AZ and its long-term effects on fish health and growth also require further investigation. Future studies should aim to validate these findings in diverse aquaculture settings and explore the scalability of AZ supplementation for widespread application in industry.

## Data Availability

All data supporting the findings of this study are available within the manuscript, figures, and tables.
